# A Sensitive and Comprehensive LC‐MS/MS Method for the Analysis of Hallucinogens, Synthetic Cathinones, and Synthetic Cannabinoids in Urine Samples

**DOI:** 10.1002/jms.5178

**Published:** 2025-09-10

**Authors:** Bruno Pereira dos Santos, Letícia Birk, Vítor Camargo Pôrto, Sabrina Nunes Nascimento, Viviane Cristina Sebben, Sarah Eller, Tiago Franco de Oliveira

**Affiliations:** ^1^ Graduate Program in Health Sciences Federal University of Health Sciences of Porto Alegre (UFCSPA) Porto Alegre Rio Grande do Sul Brazil; ^2^ Toxicological Information Center of Rio Grande do Sul (CIT‐RS) Rio Grande do Sul State Center for Health Surveillance (CEVS) Porto Alegre Rio Grande do Sul Brazil

**Keywords:** drug analysis, LC‐MS/MS, new psychoactive substances, urine

## Abstract

The laboratory analysis of new psychoactive substances and related drugs is crucial for accurate clinical and forensic diagnosis of poisonings. Given this, a new LC‐MS/MS method for analyzing hallucinogens, synthetic cathinones, and synthetic cannabinoids in urine was developed. Urine samples were extracted using a liquid–liquid extraction protocol optimized via a multivariate experimental design. An aliquot of 400 μL of urine was enzymatically hydrolyzed with β‐glucuronidase and extracted with 700 μL of ethyl acetate. The resulting extracts were analyzed using LC‐MS/MS, with a total chromatographic run time of 8 min. The method was validated according to the ANSI/ASB Standard 036 guideline and was applied to 24 samples from suspected poisoning cases. The lower limits of quantification ranged from 0.1 to 1 ng/mL. Within‐run and between‐run precision (CV) were < 16%, and bias ranged from −12.8% to 19.8%. Nine of the 20 analytes investigated showed significant ionization suppression or enhancement (> 25%). Only two analytes (2C‐E and 2‐oxo‐3‐OH‐LSD) had a recovery rate lower than 70%. Among the 24 analyzed urine samples, one tested positive for 25B‐NBOH, one for LSD, and two for 2‐oxo‐3‐OH‐LSD. The developed method enables the simultaneous quantification of 20 illicit drugs, serving as an efficient diagnostic tool for clinical and forensic laboratories.

## 1 Introduction

New psychoactive substances (NPS) represent a growing public health concern due to their potentially higher toxicity compared to classic drugs of abuse, their dynamic distribution market and easy accessibility, and their frequent absence from routine drug screening methods [1, 2]. As of June 2025, a total of 1343 NPS have been reported globally [3]. The traditional definition of NPS, as established by the United Nations Office on Drugs and Crime (UNODC), includes all substances of abuse not listed in the 1961 Single Convention on Narcotic Drugs or the 1971 Convention on Psychotropic Substances [1, 3].

Among the main groups of NPS are synthetic cathinones, synthetic phenethylamines, synthetic cannabinoid receptor agonists (SCRAs), and synthetic opioids [2]. These compounds, often designed to mimic the effects of controlled substances, pose significant challenges in clinical and forensic toxicology, including nonspecific clinical presentations, reliance on highly sensitive and comprehensive analytical methods, and limited availability of evidence‐based treatment and management protocols for intoxicated patients [4, 5]. Common clinical manifestations of NPS use include cardiovascular disturbances, central nervous system and respiratory depression, hallucinations, delirium, psychosis, and anxiety [6]. The use of NPS, either alone or in combination with other NPS or additional substances, can lead to life‐threatening or fatal outcomes [7].

Analytical approaches for the detection of NPS have significantly improved in recent years. A diverse range of validated methods for the determination of NPS in whole blood [8, 9, 10], plasma [11, 12], urine [13, 14], and hair [15] has been described in the literature. Typically, NPS are present in biological matrices at low concentrations. In addition, the development of comprehensive analytical methods that enable the simultaneous detection of multiple substances or classes is essential, given the large number of NPS currently available to the population. To overcome these challenges, the use of highly sensitive and selective techniques, such as liquid chromatography–tandem mass spectrometry (LC‐MS/MS), is recommended [16]. The implementation of LC‐MS/MS‐based methods in clinical and forensic laboratories is critical for strengthening NPS surveillance, improving patient management, and supporting forensic investigations [5].

Urine analysis allows a broader detection window, increasing the likelihood of obtaining a positive result. Urine is also considered a cleaner biological matrix, with fewer interferents, ample sample volumes (~20–30 mL), higher analyte concentrations, and frequent availability in clinical and forensic cases [17, 18]. An important aspect to consider in urine analysis is biotransformation, as it may be necessary to target the biotransformation products rather than unchanged substances. Furthermore, a hydrolysis step may be required for drugs that are conjugated with polar groups [17, 19, 20, 21].

Given this, this study aimed to develop and validate a quantitative LC‐MS/MS method for the simultaneous analysis of 20 drugs of abuse, including hallucinogens (2C‐E, 25B‐NBOH, 25B‐NBOMe, 25C‐NBOMe, 25E‐NBOH, 25I‐NBOH, 25I‐NBOMe, 2‐oxo‐3‐OH‐LSD, and LSD), synthetic cathinones (4‐chlor‐dimethylcathinone, dipentylone, ethylone, eutylone, mephedrone, methylone, N‐butylpentylone, N‐ethylheptedrone, and pentylone), and synthetic cannabinoids (ADB‐BUTINACA and ADB‐FUBINACA) in urine samples from clinical poisoning cases.

## 2 Materials and Methods

### 2.1 Chemicals and Reagents

LC‐MS‐grade acetonitrile, ethyl acetate, hexane, methyl tert‐butyl ether (MTBE), and 99% formic acid were acquired from Merck (Darmstadt, Germany). Standard solutions of 25B‐NBOMe, 25C‐NBOMe, 25I‐NBOH, 25I‐NBOMe, ethylone, lysergic acid diethylamide (LSD), mephedrone, methylone, and pentylone (1 mg/mL) were purchased from Cerilliant (Round Rock, TX, USA). 25B‐NBOH, 2C‐E, 25E‐NBOH, 4‐chloro‐N,N‐dimethylcathinone (4‐CDC), ADB‐BUTINACA, ADB‐FUBINACA, dipentylone, N‐butylpentylone, and N‐ethylpentedrone (1–5 mg total) were purchased from Cayman Chemical (Ann Arbor, MI, USA). Standard solutions of 2‐oxo‐3‐OH‐LSD (100 μg/mL) and eutylone (1 mg/mL) were acquired from LGC Standards (Manchester, NH, USA). The internal standard (IS) solutions MDEA‐*d*_6_ and PCP‐*d*_5_ (1 mg/mL) were also purchased from LGC Standards (Manchester, NH, USA). Ammonium acetate, sodium hydroxide, and β‐glucuronidase (type B‐1, from bovine liver) were purchased from Sigma‐Aldrich (St. Louis, MO). Ultrapure water was supplied by a Milli‐Q system (Millipore, Billerica, MA, USA).

### 2.2 Solutions

A working solution containing all analytes was prepared in acetonitrile at a concentration of 250 ng/mL. When not in use, the solutions were stored at −20°C. Blank urine samples were spiked with the working solution for the optimization and validation procedures. Drug‐free samples were collected from the researchers and previously analyzed to confirm the absence of target analytes. Both blank and spiked urine samples were stored in a freezer (−20°C) when not in use. The IS solution, containing MDEA‐*d*_6_ and PCP‐*d*_5_, was prepared in acetonitrile at a concentration of 400 ng/mL.

### 2.3 LC‐MS/MS Settings

The analyses were performed using a liquid chromatographic system coupled to a triple quadrupole mass spectrometer (LC‐MS/MS), model LCMS‐8045 (Shimadzu, Kyoto, Japan). The chromatographic separation was achieved using a Raptor Biphenyl column (50 × 3.0 mm, 2.7 μm particle size) from Restek (Bellefonte, PA, USA). Ultrapure water (A) and acetonitrile (B), both acidified with 0.1% (*v/v*) formic acid, were used as mobile phases, with a flow rate of 0.5 mL/min. The column oven was set at 50°C. The elution gradient used is described as follows: 0.0–0.25 min, 5%–5% of B; 0.25–4.8 min, 15%–50% of B%; 4.8–5.5 min, 50%–100% of B; 5.5–6.5 min, 100% of B; 6.5–6.6 min, 100%–5% of B; 6.6–8.0 min, 5% of B. The total chromatographic run time was 8 min.

**FIGURE 1:**
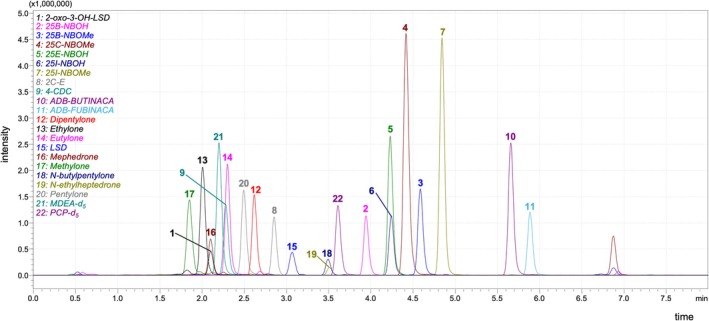
Chromatogram of all analytes included in this study, at medium QC level (12 ng/mL).

Mass spectral data were acquired using electrospray ionization (ESI) in positive mode. The mass spectrometer settings used were as follows: heat block temperature, 400°C; capillary voltage, 1.5 kV; nebulizer gas (N_2_) flow, 3.0 L/min; drying gas (N_2_) flow, 10 L/min; desolvation line temperature, 250°C; and collision‐induced dissociation (CID) gas pressure (Ar), 230 kPa. Analyses were performed in multiple reaction monitoring (MRM) mode. For each analyte, three MRM transitions were monitored: one for quantification and two for confirmation. MRM transitions, collision energies, and retention time for each substance are presented in Table 1. Figure 1 shows a representative chromatogram of all analytes extracted from a spiked urine sample at 12 ng/mL concentration. All data were processed using the LabSolutions software (Shimadzu, Kyoto, Japan).

**TABLE 1:** Analytes and their respective mass spectrometry conditions, retention time, and internal standard (IS).

Analyte	MRM transitions (*m/z*)	Dwell time (s)	Q1 pre bias (V)	ce (eV)	Q3 pre bias (V)	Retention time (min)	IS
2C‐E	210.0 → 193.1	1.0	10	14	21	2.81	PCP‐*d*_5_
	210.0 → 178.1	1.0	14	20	17		
	210.0 → 163.0	1.0	10	29	30		
25B‐NBOH	366.1 → 243.0	1.0	13	24	25	3.95	PCP‐*d*_5_
	366.1 → 107.0	1.0	13	28	18		
	366.1 → 260.1	1.0	13	12	17		
25B‐NBOMe	380.0 → 121.1	1.0	13	22	22	4.59	PCP‐*d*_5_
	380.0 → 91.1	1.0	11	45	16		
	380.0 → 93.1	1.0	13	32	16		
25C‐NBOMe	336.1 → 121.1	1.0	12	21	23	4.42	PCP‐*d*_5_
	336.1 → 91.1	1.0	12	48	15		
	336.1 → 93.1	1.0	12	40	16		
25E‐NBOH	316.0 → 193.1	1.0	21	21	18	4.25	PCP‐*d*_5_
	316.0 → 107.1	1.0	11	31	18		
	316.0 → 178.1	1.0	21	31	17		
25I‐NBOH	414.0 → 290.9	1.0	15	24	30	4.25	PCP‐*d*_5_
	414.0 → 107.1	1.0	15	31	18		
	414.0 → 308.0	1.0	15	14	20		
25I‐NBOMe	428.0 → 121.1	1.0	15	24	21	4.90	PCP‐*d*_5_
	428.0 → 91.1	1.0	15	53	15		
	428.0 → 93.1	1.0	15	46	17		
2‐Oxo‐3‐OH‐LSD	356.0 → 237.1	1.0	24	25	23	2.11	MDEA‐*d*_5_
	356.0 → 222.0	1.0	24	34	22		
	356.0 → 265.1	1.0	24	19	28		
4‐CDC	212.0 → 139.0	1.0	19	20	21	2.28	MDEA‐*d*_5_
	212.0 → 167.0	1.0	19	15	25		
	212.0 → 103.0	1.0	18	25	14		
ADB‐BUTINACA	331.2 → 201.1	1.0	12	27	20	5.67	PCP‐*d*_5_
	331.2 → 314.2	1.0	12	10	21		
	331.2 → 286.1	1.0	12	16	18		
ADB‐FUBINACA	383.1 → 338.2	1.0	22	16	21	5.89	PCP‐*d*_5_
	383.1 → 366.2	1.0	13	10	24		
	383.1 → 253.1	1.0	14	26	22		
Dipentylone	250.0 → 100.1	1.0	17	23	18	2.62	MDEA‐*d*_5_
	250.0 → 205.0	1.0	16	15	21		
	250.0 → 175.1	1.0	17	20	17		
Ethylone	222.0 → 174.1	1.0	20	20	20	2.02	MDEA‐*d*_5_
	222.0 → 204.1	1.0	16	14	20		
	222.0 → 146.1	1.0	16	29	14		
Eutylone	236.0 → 188.1	1.0	20	20	20	2.30	MDEA‐*d*_5_
	236.0 → 218.0	1.0	20	15	20		
	236.0 → 189.1	1.0	20	25	20		
LSD	324.2 → 223.0	1.0	12	26	22	3.04	MDEA‐*d*_5_
	324.2 → 281.1	1.0	12	19	30		
	324.2 → 207.0	1.0	21	45	20		
Mephedrone	178.0 → 160.1	1.0	20	15	20	2.10	MDEA‐*d*_5_
	178.0 → 145.1	1.0	20	16	20		
	178.0 → 144.1	1.0	18	15	20		
Methylone	208.1 → 160.1	1.0	20	20	20	1.86	MDEA‐*d*_5_
	208.1 → 132.1	1.0	15	22	23		
	208.1 → 190.1	1.0	15	12	17		
N‐Butylpentylone	278.1 → 260.2	1.0	20	15	20	3.46	MDEA‐*d*_5_
	278.1 → 230.1	1.0	20	20	20		
	278.1 → 188.0	1.0	20	20	20		
N‐Ethylheptedrone	234.1 → 216.1	1.0	20	15	20	3.48	MDEA‐*d*_5_
	234.1 → 91.1	1.0	20	30	20		
	234.1 → 146.1	1.0	20	20	20		
Pentylone	236.1 → 188.1	1.0	20	20	20	2.49	MDEA‐*d*_5_
	236.1 → 218.1	1.0	20	14	20		
	236.1 → 131.1	1.0	18	40	22		
MDEA‐*d*_5_	213.1 → 163.1	1.0	20	15	20	2.18	—
	213.1 → 105.1	1.0	20	30	20		
	213.1 → 135.1	1.0	19	23	20		
PCP‐*d*_5_	249.3 → 96.1	1.0	20	32	20	3.61	—
	249.3 → 164.2	1.0	20	18	20		
	249.3 → 86.1	1.0	20	22	20		

Abbreviations: ce, collision energy; IS, internal standard; MRM, multiple reaction monitoring.

### 2.4 Sample Procedure

An aliquot of 400 μL of urine and 20 μL of the IS solution (400 ng/mL) were added to a polypropylene microtube. The sample was hydrolyzed by the addition of 10 μL of β‐glucuronidase (400 U/μL) and 50 μL of acetate buffer (0.2 mol/L, pH 5.2), and the mixture was heated at 55°C for 60 min. After hydrolysis, the sample was cooled to room temperature. Then, 100 μL of NaOH solution (0.1 mol/L) and 700 μL of ethyl acetate were added. The mixture was vortexed for 10 s and centrifuged for 6 min at 9000 rpm. Subsequently, 650 μL of the organic phase was transferred to a new tube and dried under nitrogen flow at 50°C. The dried extract was reconstituted with 30 μL of ACN:water (3:1, *v/v*) with 0.1% (*v/v*) formic acid. Finally, 1 μL was injected into the LC‐MS/MS.

### 2.5 Optimization of Sample Extraction

Sample preparation is an essential step for obtaining bioanalytical methods with reduced matrix interferents and improved sensitivity. To achieve this, proper optimization must be carried out to ensure that the best conditions for the parameters involved in the extraction process [22]. In this study, a multivariate experimental approach was employed to improve the responses of variables that can influence the extraction efficiency. Initially, a fractional factorial design with five factors (2^5–1^) was conducted to evaluate the following variables: solvent volume, sample volume, NaOH concentration, use of salting‐out, and extraction time. For each variable, two values (low and high) were defined, totaling sixteen runs. The complete experiment matrix is presented in Table S1. Subsequently, variables considered significant, when applicable, were further evaluated. A Doehlert design was employed to assess the optimal combination of solvent and sample volumes. Sample volume was optimized at five levels (100–400 μL), whereas solvent volume was evaluated in three levels (200–800 μL). The complete Doehlert experimental matrix is provided in Table S2. Next, the composition of the extractor solvent was investigated using three different solvents: ethyl acetate, hexane, and MTBE. Extraction was performed with both pure solvents and their mixtures, with ratios detailed in Table S3. All optimization experiments were conducted using drug‐free urine samples spiked with analytes at a concentration of 15 ng/mL. In both the fractional factorial and Doehlert designs, MTBE was fixed as the extraction solvent. The geometric mean of the absolute chromatographic area of all analytes was used to assess the response of the experiments. Optimization data were analyzed using Statistica 10.0 software (Statsoft, Tulsa, OK, USA) and statistically evaluated using the analysis of variance (ANOVA).

### 2.6 Bioanalytical Validation

Bioanalytical validation was performed in accordance with the Standard Practices for Method Validation in Forensic Toxicology (ANSI/ASB Standard 036 guideline), from the American Academy of Forensic Science (AAFS), as well as other international recommendations [23, 24]. The evaluated parameters included lower limit of quantification (LLOQ), interference studies, linearity, precision, bias, matrix effects, extraction recovery, dilution integrity, and processed sample stability.

The LLOQ was determined by analyzing fortified urine samples at decreasing concentrations (1.0, 0.5, and 0.1 ng/mL). Triplicate samples from three drug‐free individuals were extracted and analyzed, totaling nine evaluations. The LLOQ was defined as the lowest concentration level that presented a coefficient of variation (CV%) below 20%.

Interference studies were conducted to assess potential false signals from different urine matrices and substances that may be present in the sample. First, endogenous interference was evaluated by analyzing 10 negative urine samples from different individuals. Second, exogenous interference was assessed by adding compounds commonly found in urine samples, including cocaine and its metabolites, amphetamine‐type stimulants, cannabinoids, benzodiazepines, opioids, antidepressants, antipsychotics, and antiepileptics. Lastly, potential interference from stable isotope–labeled IS was investigated by analyzing a negative sample containing the IS solution and a fortified sample without IS addition.

To assess the linearity, calibration curves were constructed with six calibrators, ranging from the LLOQ to 25 ng/mL. The calibrators were prepared by spiking drug‐free urine samples with the working solution. All curves were required to present coefficients of determination (*R* ^2^) greater than 0.99 to be considered linear. Furthermore, Fisher's *F*‐test was used to assess the presence of heteroscedasticity. If detected, a weighted least squares model was applied using weight factors as 1/x and 1/x^2^. The optimal weighting scheme was selected based on the model yielding the lowest sum of residuals. After analysis of the upper limit of quantification (25 ng/mL), carryover was evaluated by injecting three blank samples. The method was considered free of carryover if the analyte signal in the blank samples was lower than the signal at the LLOQ.

To assess the precision and bias of the method, fortified urine samples were evaluated at three quality control (QC) concentrations: low (0.3 or 1.5 ng/mL), medium (12 ng/mL), and high (22 ng/mL). Each QC level was analyzed in triplicate within a single analytical run. Five runs were performed for each QC level, totaling 15 analyses per level. Precision was assessed by calculating within‐run and between‐run coefficients of variation (CV%) using ANOVA. According to the ANSI/ASB guideline, acceptable CV% values should be equal to or less than 20%. The same experiments were used to evaluate method bias, which was calculated as the difference between the measured and theoretical concentrations, with an acceptable range of ±20%. Additionally, dilution integrity was evaluated using a 1:20 (v/v) dilution factor to verify precision and bias when sample dilution is required due to analyte concentrations exceeding 25 ng/mL. For this purpose, fortified samples at 300 ng/mL were diluted with ultrapure water and analyzed. Acceptance criteria for this test were CV% ≤ 20% and bias within ±20%.

Matrix effect (ME) and extraction recovery were evaluated according to the procedure described by Matuszewski et al. [24]. For this purpose, three sets of six samples from different individuals were analyzed: Set A, neat solution; Set B, urine samples spiked before extraction; and Set C, urine samples spiked after extraction. The ME% was calculated by dividing Set C by that of Set A and multiplying by 100. The CV% of ME% across the six individual samples was required to be below 20%. Extraction recovery was calculated by dividing Set B by Set C and multiplying by 100. Both ME and extraction recovery were evaluated at low and high QC concentrations.

Processed sample stability was evaluated by analyzing six samples at time zero and again after 24 h of storage in the LC‐MS/MS autosampler maintained at 15°C. This parameter was assessed at all QC levels, and results were expressed as the percentage of the concentration at 24 h relative to the concentration at time zero.

### 2.7 Application to Urine Samples From Poisoning Cases

As proof of the method's applicability, 24 urine samples from poisoning cases attended by the Toxicological Information Center of Rio Grande do Sul (CIT‐RS), southern Brazil, were analyzed using the validated method. This study was approved by the Ethics Committee for Human Research at the Federal University of Health Sciences of Porto Alegre (UFCSPA), Brazil, and by the Ethics Committee for Human Research of the School of Public Health, State Health Department of Rio Grande do Sul, Brazil (CAAE 31992920.3.0000.5345 and CAAE 31992920.3.3001.5312).

## 3 Results

### 3.1 Extraction Optimization

**FIGURE 2:**
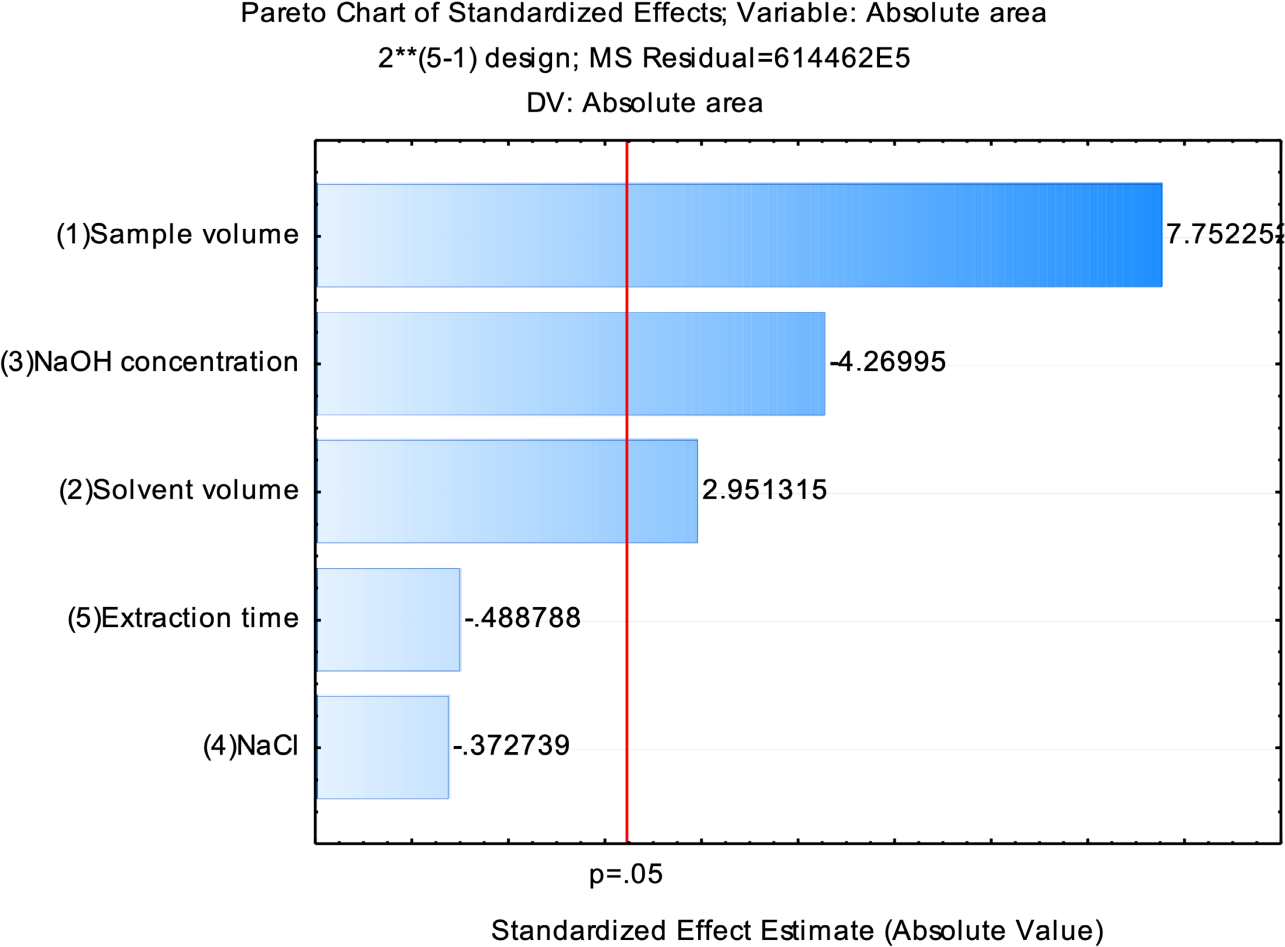
Pareto chart of effects from the variables evaluated in the fractional factorial design (2^5–1^). All variables that crossed the red line (*p* = 0.05) were considered statistically significant, that is, these variables are able to influence the analytical response.

In the fractional factorial design (2^5–1^), three variables (sample volume, solvent volume, and NaOH concentration) had a significant effect (*p* < 0.05) on the analytical response. Extraction time and the addition of NaCl were not significant, with *p* values of 0.6355 and 0.7171, respectively. These results can be visualized in the Pareto chart of effects (see Figure 2). All variables that crossed the red significance threshold line (*p* = 0.05) were considered statistically significant.

**FIGURE 3:**
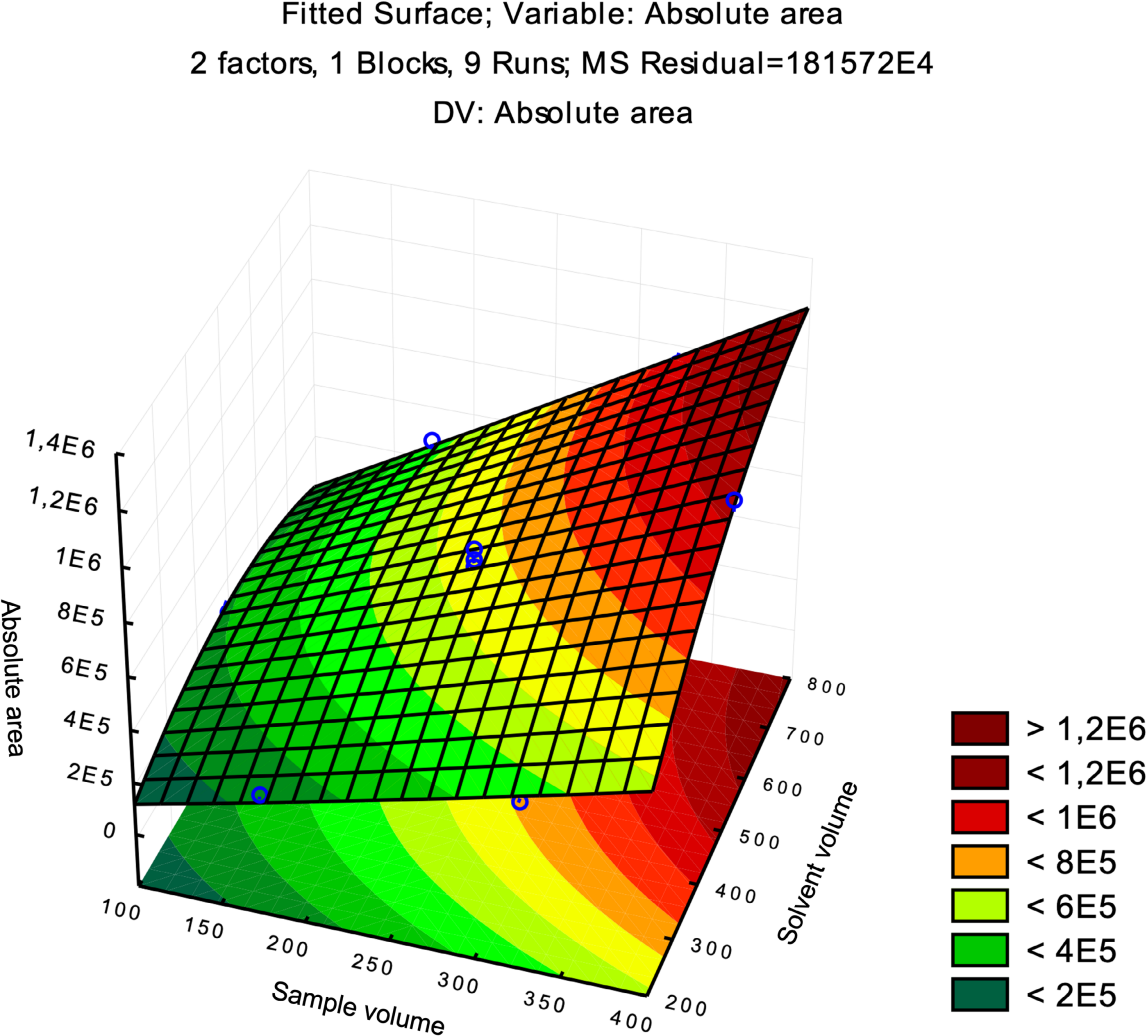
Surface response graph from Doehlert design for the evaluation of sample and extractor solvent volumes.

**FIGURE 4:**
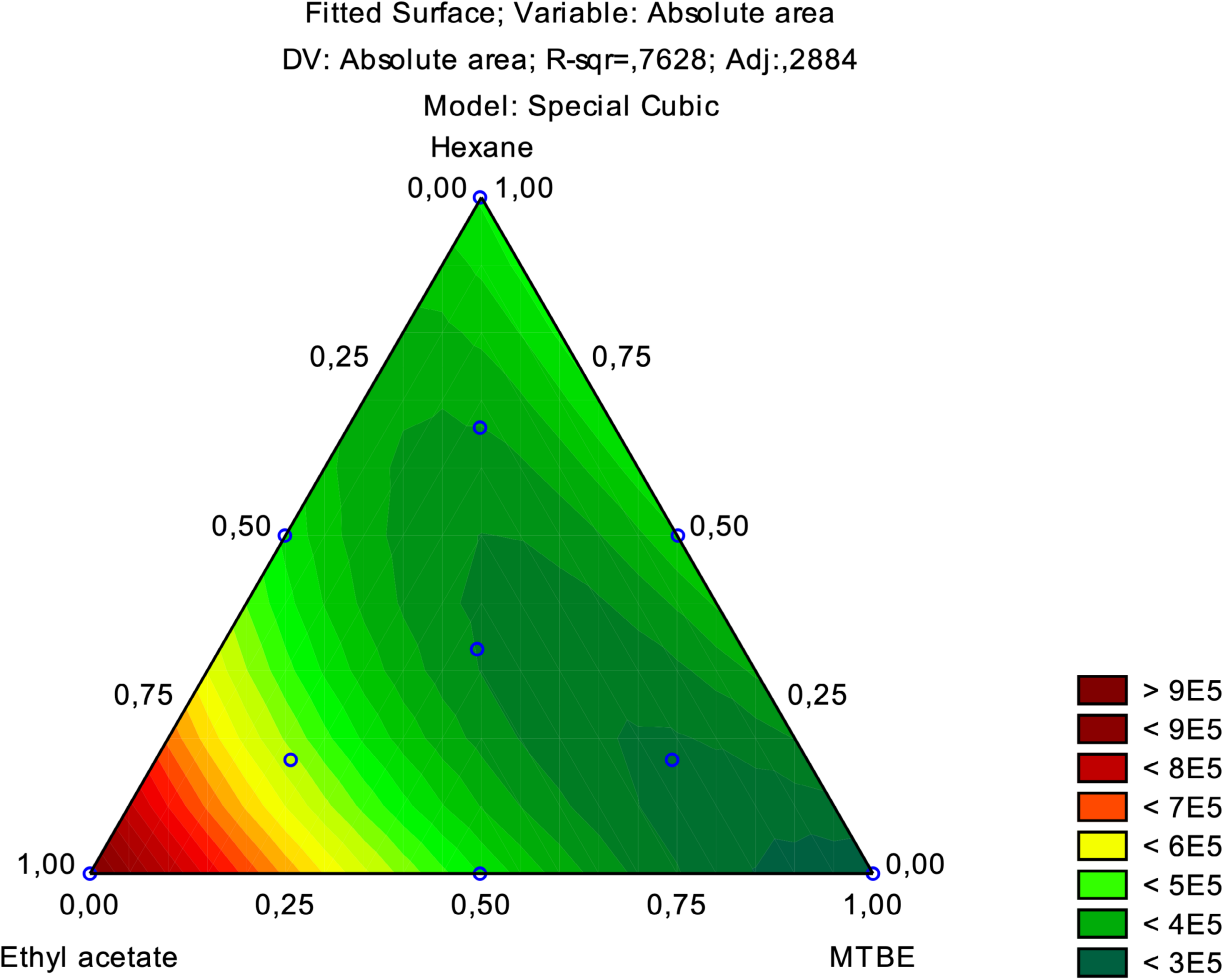
Surface response from simplex‐centroid design for the optimization of the type of extractor solvent.

The subsequent optimization focused exclusively on sample volume and solvent volume, as low NaOH concentrations had already been evaluated in the fractional factorial design. The standardized effect value for NaOH concentration indicates a trend toward better responses at the lowest tested level, corresponding to 0.1 mol/L NaOH. The Doehlert design demonstrated a more sensitive analytical response when using the highest volumes of both sample and extraction solvent. This result was expected, because the larger the sample volume, the greater the amount of analyte available to be extracted. The surface response plot is shown in Figure 3, where the red region represents the highest response (geometric mean of absolute areas). The *R* ^2^ value of the model was 0.9909. Finally, the simplex‐centroid optimization indicates neat ethyl acetate as the most suitable extractor solvent, as shown in Figure 4. The obtained *R* ^2^ for the design was 0.7628.

### 3.2 Bioanalytical Validation

The complete validation data is presented in Table 2. The LLOQ ranged from 0.1 to 1.0 ng/mL, with CV% values below 20% between the nine replicates. All analytes showed acceptable linearity, with *R* ^2^ values greater than 0.9918. Except for 2‐oxo‐3‐OH‐LSD, the calibration curves exhibited heteroscedasticity, requiring the application of weight factors. The weighting schemes applied were 1/x and 1/x^2^, both commonly used in analytical toxicology. No carryover effect was observed following the injection of the upper limit of quantification. During the interference assessment, no peaks were observed at the retention times of the analytes or IS, either in the analyses of urine samples fortified with exogenous compounds or in the analysis of drug‐free urine samples from different sources, confirming the selectivity of the method. Additionally, no interference from the stable isotope–labeled ISs was identified.

**TABLE 2:** Results for LLOQ, linearity, precision, bias, matrix effect, and extraction recovery parameters.

Analyte	LLOQ (ng/mL)	Linearity		Low QC			Medium QC			High QC			Matrix effect (%)	Extraction recovery (%)
		*R* ^2^	Weighting factor	Within‐run precision (CV%)	Between‐run precision (CV%)	Bias (%)	Within‐run precision (CV%)	Between‐run precision (CV%)	Bias (%)	Within‐run precision (CV%)	Between‐run precision (CV%)	Bias (%)		
Hallucinogens														
2C‐E	0.5	0.9999	1/x	11.0	5.7	−12.8	7.0	4.3	−8.5	5.6	3.9	−11.1	147.7	60.2
25B‐NBOH	0.5	0.9999	1/x^2^	7.9	4.6	2.1	5.8	4.5	1.9	6.0	3.5	−2.4	117.8	86.0
25B‐NBOMe	0.1	0.9939	1/x^2^	15.8	8.7	11.4	5.7	4.1	10.4	7.7	4.6	12.9	153.0	80.8
25C‐NBOMe	0.1	0.9935	1/x^2^	7.2	5.8	7.2	13.1	7.3	18.5	5.4	3.0	3.1	172.6	83.0
25E‐NBOH	0.1	0.9996	1/x^2^	10.6	6.3	−6.7	5.6	3.9	7.2	5.1	2.5	2.1	178.5	89.6
25I‐NBOH	0.5	0.9981	1/x	12.0	6.8	−7.9	5.0	3.5	12.5	9.4	4.6	18.3	157.2	94.0
25I‐NBOMe	0.1	0.9999	1/x^2^	9.7	5.1	4.7	9.7	5.0	19.8	8.4	4.1	16.7	164.9	83.8
2‐Oxo‐3‐OH‐LSD	0.5	0.9995	1	7.6	4.2	−1.2	7.4	4.7	−6.7	9.3	5.3	−6.0	104.5	38.9
LSD	0.5	0.9918	1/x	11.6	7.0	10.1	10.9	5.6	13.5	6.1	4.2	0.5	277.7	75.9
Synthetic cannabinoids														
ADB‐BUTINACA	0.5	0.9977	1/x^2^	4.0	2.6	0.7	5.9	4.2	4.8	9.7	4.8	3.5	114.7	80.5
ADB‐FUBINACA	0.5	0.9962	1/x	10.6	6.1	7.6	4.4	2.6	14.5	8.2	4.5	9.2	106.8	81.5
Synthetic cathinones														
4‐CDC	0.5	0.9930	1/x	12.5	6.7	0.1	9.2	7.0	6.7	8.0	5.4	2.4	53.7	94.7
Dipentylone	0.5	0.9999	1/x^2^	15.3	7.7	−9.1	8.4	5.4	0.9	6.9	3.7	−2.2	90.7	91.6
Ethylone	0.5	0.9995	1/x^2^	6.1	4.1	3.8	7.9	4.0	6.6	4.2	3.0	2.2	72.8	75.6
Eutylone	0.5	0.9982	1/x^2^	5.5	4.1	−3.0	7.1	4.3	−0.7	4.8	2.8	−9.8	96.3	92.7
Mephedrone	1	0.9998	1/x^2^	14.1	7.9	−10.4	9.4	4.9	6.2	4.2	2.5	3.9	116.5	76.5
Methylone	0.5	0.9998	1/x	7.2	4.4	−8.3	4.7	3.7	0.7	5.3	2.8	1.7	89.4	79.9
N‐Butylpentylone	1	0.9962	1/x^2^	13.9	9.5	3.3	8.5	4.2	13.8	7.0	4.0	12.8	124.9	91.8
N‐Ethylheptedrone	1	0.9989	1/x^2^	11.0	5.8	−0.7	8.5	4.5	3.9	4.9	3.8	6.8	87.9	89.5
Pentylone	0.5	0.9998	1/x^2^	8.1	5.1	−2.1	5.6	3.1	4.9	4.4	2.6	5.6	82.7	88.2

Abbreviations: CV, coefficient of variation; LLOQ, lower limit of quantification; QC, quality control; *R* ^2^, coefficient of determination.

The obtained within‐run and between‐run precision values were below 20% for all QC concentrations, in accordance with the ANSI/ASB guideline [23]. Within‐run precision CVs ranged from 4.0% to 15.8%, corresponding to the low QCs of ADB‐BUTINACA and 25B‐NBOMe, respectively. Between‐run precision CVs ranged from 2.5% (high QCs of 25E‐NBOH and mephedrone) to 9.5% (low QC of N‐butylpentylone). The observed biases ranged from −12.8% to 19.8%, associated with the low QC of 2C‐E and the medium QC of 25I‐NBOMe, respectively. Dilution integrity results showed within‐run and between‐run CV% and bias values within the ±20% acceptance range.

The ME varied considerably among analytes, ranging from 53.7% to 277.7%. Nine substances exhibited significant ME (defined as < 75% or > 125%). In the ME assessment of 4‐CDC and ethylone, ion suppression was observed, whereas ion enhancement was detected for 2C‐E, 25B‐NBOMe, 25C‐NBOMe, 25E‐NBOH, 25I‐NBOH, 25I‐NBOMe, and LSD. Despite ME values falling outside the 75%–125% range, all CV% values between replicates were below 20%, indicating the consistency and reproducibility of these effects. Extraction recovery rates were generally high, with only two analytes exhibiting values below 70%. The analytes with low or moderate recovery—2‐oxo‐3‐OH‐LSD (38.9%) and 2C‐E (60.2%)—are more polar compared to the other compounds.

Additionally, processed sample stability over 24 h ranged from 73.2% to 126.4%. Only three analytes exhibited notable instability (outside the ±25% range): the medium QC of 2‐oxo‐3‐OH‐LSD and the low QC levels of N‐butylpentylone and pentylone. Complete processed sample stability data are provided in Table S4.

### 3.3 Analysis of Samples From Poisoning Cases

**FIGURE 5:**
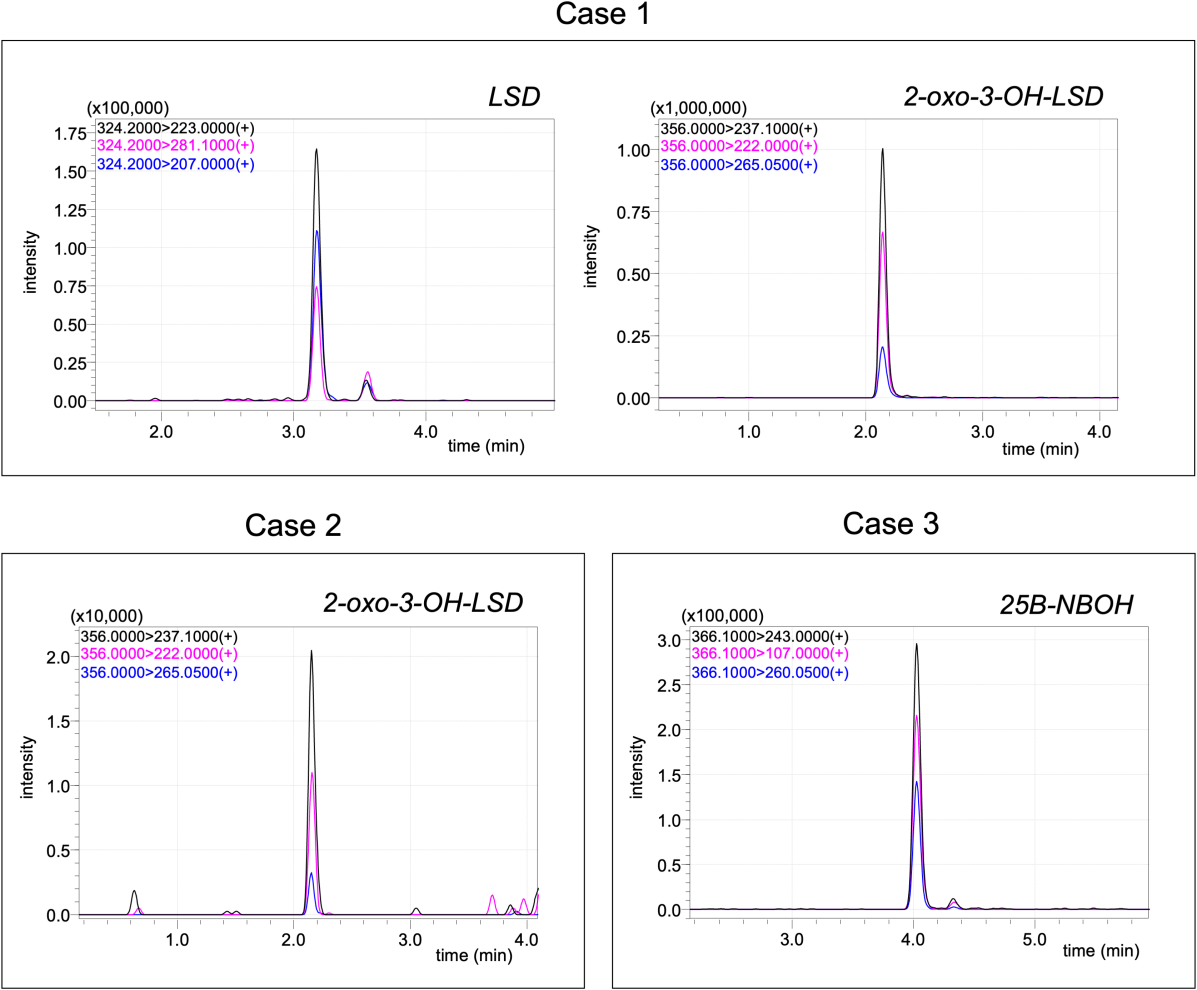
MRM chromatogram of three urine samples with a positive result for at least one analyte.

Of the 24 urine samples analyzed from suspected poisoning cases, three tested positive for at least one target compound. Case 1 was positive for LSD (73.8 ng/mL) and its metabolite, 2‐oxo‐3‐OH‐LSD (> 300 ng/mL). Case 2 was positive for 2‐oxo‐3‐OH‐LSD alone (3.8 ng/mL). Finally, Case 3 was positive for 25B‐NBOH, with a concentration of 8.4 ng/mL. MRM chromatograms of the three positive cases are shown in Figure 5.

## 4 Discussion

This study presented a sensitive and targeted analytical method for the detection of 20 drugs in urine, including NPS from different chemical classes. The NPS panel was defined based on the most relevant substances circulating in Brazilian territory in recent years. Historically, synthetic cathinones and synthetic phenethylamines have been the most frequently encountered NPS in Brazil, according to national drug seizure data [25]. Since the early 2020s, SCRAs have gained increasing prominence in the Brazilian context [26, 27]. Although LSD and its main urinary metabolite, 2‐oxo‐3‐OH‐LSD, are not classified as NPS, they were included in the method due to the common substitution of LSD with NBOMe and NBOH derivatives, particularly in blotter papers or powders [28, 29]. In the state of Rio de Janeiro, analysis of 3899 blotter papers seized between 2006 and 2019 identified 25I‐NBOH as the most prevalent substance (41.3%), followed by 25I‐NBOMe (16.6%) and LSD (14.2%) [30]. In another Brazilian state, Santa Catarina, blotter papers seized between 2011 and 2017 were analyzed by GC‐MS, and the presence of LSD in positive samples per year ranged from 0.12% to 100% [31].

A major challenge in the simultaneous analysis of diverse compounds such as NPS is their wide chemical variability. This complexity makes it difficult to establish a single sample preparation procedure capable of efficiently extracting structurally distinct analytes while maintaining high sensitivity and precision. The use of multivariate experimental design enabled the simultaneous assessment of multiple variables and the selection of optimal extraction conditions for all target compounds included in the study. Additionally, the structural similarity between certain substances, particularly positional isomers, complicates their detection and quantification. In this study, pentylone and eutylone shared the same MRM transitions. Therefore, chromatographic separation was essential for distinguishing them. The gradient employed successfully achieved baseline separation between the two synthetic cathinones, with retention times of 2.30 min for eutylone and 2.49 min for pentylone. Despite this level of resolution, the chromatographic method remains relatively fast, being shorter than other LC‐MS methods for NPS analysis reported in the literature [10, 13, 32, 33].

In urine analysis, hydrolysis is a fundamental step for the cleavage of the analyte with polar groups, such as glucuronic acid. Therefore, in this study, the samples were incubated with the β‐glucuronidase enzyme to obtain the analytes in their unconjugated form. Several studies indicate that some of the NPS included in this work can be excreted conjugated with glucuronic acid [34, 35, 36, 37, 38]. Pelletier et al. analyzed a urine sample from a poisoning case involving 25E‐NBOH, and the glucuronidated metabolite was the most intense, with a relative response to the unchanged 25E‐NBOH higher than 2000% [39]. Xiang et al. recommended the use of β‐glucuronidase for urine analysis of NBOH and NBOMe compounds to avoid false negative results [38]. Nonetheless, the absence of NPS metabolites in the current method should be acknowledged as an important limitation of this study. This is an important point to consider, as the concentration of metabolites in urine tends to be higher than that of the unchanged molecule, as can be seen in Case 1. The inclusion of metabolites helps avoid false negatives, as well as increasing the drug's detection window.

The developed method demonstrated high sensitivity, with LLOQs equal to or lower than 1 ng/mL. Some NPS, mainly SCRAs and phenethylamines, require enhanced sensitivity, as they are typically present in low concentrations in biological samples [16]. The obtained LLOQs are comparable to or inferior to the reported limits of quantification from other published methods [10, 13, 14, 32, 40, 41, 42, 43, 44].

The ME results indicated an intense ionization enhancement for some analytes, mainly phenethylamines. Previous studies have not reported altered ME values for these substances. Ferrari Júnior and Caldas described ME values for between 75.4% and 109.3% for LSD, NBOMe, and NBOH compounds [10]. Fan et al. reported normal ME values (86% to 119%) for 4‐CDC, ethylone, 25B‐NBOMe, 2C‐E, and 25I‐NBOMe [13, 41]. On the other hand, Fabris et al. verified an unusual ionization enhancement for synthetic cathinones when using a dispersive liquid–liquid microextraction protocol [45]. In this study, the CV% of ME replicates was below 20%, that is, the ME, although significant, is constant. This means that the interindividual variability of urine samples affects the ME homogeneously. In this way, it can be ensured that the blank urine employed in the validation studies modulates the analytical response similarly to the urine samples to be tested and quantified. This allows the developed method to be used in the reliable quantification of the listed analytes. Better chromatographic separation to avoid coelution with endogenous interferents or more extensive sample preparation techniques (e.g., solid‐phase extraction) could result in more suitable ME values. Additionally, the use of stable isotope–labeled IS of each analyte is recommended to minimize ME on quantification methods [24, 46, 47]. However, all these procedures demand a higher analysis time or associated cost.

In the processed sample stability assessment, instability greater than 125% was verified for three analytes (LSD, N‐butylpentylone, and pentylone) in some QC levels. In this sense, the analysis of extracts after 24 h may generate increased concentrations of these substances. Therefore, immediate analysis after the extraction process is recommended, at least when these analytes are suspected to be present.

Regarding the cases with positive results, Case 1 involved a male individual of unknown age who was found unconscious at an electronic music festival. Upon admission, he was in a coma, with malignant hyperthermia, hyporeflexia, mydriasis, tachycardia, and respiratory depression. A urine sample was collected and sent to the CIT‐RS laboratory. Hours later, the patient died due to cardiorespiratory arrest. When screened for classic drugs and medications [17], the urine sample tested positive for MDA (> 10 μg/mL), MDEA (561 ng/mL), MDMA (> 10 μg/mL), ketamine (534 ng/mL), lidocaine (1.7 μg/mL), and diclofenac (81 ng/mL). Typically, LSD and its metabolite 2‐oxo‐3‐OH‐LSD are detected at low concentrations (below 25 ng/mL) in urine samples. However, elevated concentrations may occur following high‐dose LSD use [48, 49, 50]. Moreover, markedly high urinary concentrations of LSD and 2‐oxo‐3‐OH‐LSD can be observed in cases of severe poisoning or fatal outcomes. Favretto et al. reported postmortem urine concentrations of LSD and 2‐oxo‐3‐OH‐LSD of 91.0 and 430.2 ng/mL, respectively [51], which are comparable to those found in Case 1. In this case, the concomitant presence of high urinary levels of MDA and MDMA may have exerted a cumulative effect contributing to death alongside LSD.

Case 2 involved a 35‐year‐old woman, reportedly a drug user in withdrawal, though a psychiatric report raised suspicions of ongoing substance use. In this case, only the main LSD metabolite, 2‐oxo‐3‐OH‐LSD, was detected. Additional urine screening revealed the presence of MDA, MDMA, 11‐nor‐Δ^9^‐tetrahydrocannabinol carboxylic acid, 7‐aminoclonazepam, citalopram, oxazepam, and temazepam. A positive result exclusively for 2‐oxo‐3‐OH‐LSD without LSD detection can be explained by the longer detection window of this metabolite compared to the parent compound [48].

Case 3 involved a 17‐year‐old male adolescent admitted to the hospital presenting with intense agitation. The patient reported using “N‐bomb,” a common street name for substances containing NBOMe derivatives. LC‐MS/MS analysis for drugs and medications [17] also detected lidocaine, haloperidol, and promethazine, which were administered during hospital care. No other drugs of abuse were identified. Although the patient referred to NBOMe use, NBOH compounds currently represent the majority of circulating phenethylamines in Brazil [25, 52]. Ivory et al. described a case series involving 25B‐NBOH exposure, including a patient with severe agitation, as observed in Case 3. That report also mentioned acute kidney injury and rhabdomyolysis [28]. In Case 3, no renal damage was identified; however, elevated levels of total and indirect bilirubin and creatine phosphokinase (CK) were observed, suggesting hemolysis and muscle injury, likely related to the intense agitation presented by the patient.

## 5 Conclusion

In this work, a method for the analysis of 20 drugs of abuse, including hallucinogens, synthetic cathinones, and synthetic cannabinoids, was successfully developed and validated. The established protocol is simple, efficient, and suitable for detecting NPS at low concentrations. Validation followed the ANSI/ASB Standard 036 guideline, with all assessed mandatory parameters meeting the established acceptance criteria. As proof of applicability, 24 urine samples from suspected poisoning cases were analyzed, with three yielding positive results for at least one of the target substances. The proposed method demonstrates strong potential as a diagnostic tool for NPS‐related intoxications and can be readily implemented in clinical and forensic toxicology laboratories.

## Conflicts of Interest

The authors declare no conflicts of interest.

## Supporting information


**Table S1:** Complete matrix from fractional factorial design (2^5–1^) for screening of significant variables.
**Table S2:** Complete matrix from Doehlert design for solvent and sample volume optimization.
**Table S3:** Complete matrix from simplex‐centroid design for extractor solvent optimization.
**Table S4:** Processed sample stability results for each QC level.

## Data Availability

The data that support the findings of this study are available from the corresponding author upon reasonable request.

## References

[1] A. Shafi , A. J. Berry , H. Sumnall , D. M. Wood , and D. K. Tracy , “New Psychoactive Substances: A Review and Updates,” Therapeutic Advances in Psychopharmacology 10 (2020): 2045125320967197. 33414905 10.1177/2045125320967197 PMC7750892

[2] A. Y. Simão , M. Antunes , E. Cabral , et al., “An Update on the Implications of New Psychoactive Substances in Public Health,” International Journal of Environmental Research and Public Health 19 (2022): 4869. 35457736 10.3390/ijerph19084869 PMC9028227

[3] UNODC , “NPS Data Visualisations,” (2025), https://www.unodc.org/LSS/Page/NPS/DataVisualisations.

[4] I. C. Santos , D. Maia , R. J. Dinis‐Oliveira , and D. J. Barbosa , “New Psychoactive Substances: Health and Legal Challenges,” Psychoactives 3 (2024): 285–302.

[5] J. J. Miller , M. Yazdanpanah , D. A. Colantonio , D. R. Beriault , and S. R. Delaney , “New Psychoactive Substances: A Canadian Perspective on Emerging Trends and Challenges for the Clinical Laboratory,” Clinical Biochemistry 133–134 (2024): 110810. 10.1016/j.clinbiochem.2024.110810 39181179

[6] A. Peacock , R. Bruno , N. Gisev , et al., “New Psychoactive Substances: Challenges for Drug Surveillance, Control, and Public Health Responses,” Lancet 394 (2019): 1668–1684. 31668410 10.1016/S0140-6736(19)32231-7

[7] M. Kraemer , A. Boehmer , B. Madea , and A. Maas , “Death Cases Involving Certain New Psychoactive Substances: A Review of the Literature,” Forensic Science International 298 (2019): 186–267. 30925344 10.1016/j.forsciint.2019.02.021

[8] J. Ayala and S. Kerrigan , “Comprehensive Toxicological Screening of Common Drugs of Abuse, New Psychoactive Substances and Cannabinoids in Blood Using Supported Liquid Extraction and Liquid Chromatography–Quadrupole Time‐of‐Flight Mass Spectrometry,” Journal of Analytical Toxicology 47 (2023): 656–667. 37702353 10.1093/jat/bkad069

[9] L. Birk , B. P. Dos Santos , S. Eller , and T. F. De Oliveira , “A Novel Method for the Determination of Synthetic Cathinones and Related Substances in Postmortem Blood Samples Using Cork‐Based Dispersive Solid‐Phase Microextraction Prior to LC‐MS/MS Analysis,” Analytical and Bioanalytical Chemistry 417, no. 17 (2025): 3845–3855. 40369295 10.1007/s00216-025-05907-y

[10] E. Ferrari Júnior and E. D. Caldas , “Determination of New Psychoactive Substances and Other Drugs in Postmortem Blood and Urine by UHPLC–MS/MS: Method Validation and Analysis of Forensic Samples,” Forensic Toxicology 40 (2022): 88–101. 36454493 10.1007/s11419-021-00600-y

[11] J. Hwang , M. Han , S. An , J. H. Moon , G. Shim , and H. Chung , “Screening of New Psychoactive Substances in Human Plasma by Magnetic Solid Phase Extraction and LC‐QTOF‐MS,” Forensic Science International 332 (2022): 111176. 35033963 10.1016/j.forsciint.2022.111176

[12] A. L. Fabris , S. Pedersen‐Bjergaard , E. L. Øiestad , et al., “Solvent‐Free Parallel Artificial Liquid Membrane Extraction for Drugs of Abuse in Plasma Samples Using LC‐MS/MS,” Analytica Chimica Acta 1301 (2024): 342387. 38553114 10.1016/j.aca.2024.342387

[13] S.‐Y. Fan , C.‐Z. Zang , P.‐H. Shih , et al., “Simultaneous LC‐MS/MS Screening for Multiple Phenethylamine‐Type Conventional Drugs and New Psychoactive Substances in Urine,” Forensic Science International 325 (2021): 110884. 34245937 10.1016/j.forsciint.2021.110884

[14] J. Czarny , J. Musiał , J. Powierska‐Czarny , et al., “Determination of 465 Psychoactive Substances, Drugs and Their Metabolites in Urine by LC‐MS/MS,” Analytical Methods 16 (2024): 5426–5432. 39037182 10.1039/d4ay00777h

[15] R. Barone , G. Pelletti , A. Giorgetti , et al., “Validation and Application of a Method for the Quantification of 137 Drugs of Abuse and New Psychoactive Substances in Hair,” Journal of Pharmaceutical and Biomedical Analysis 243 (2024): 116054. 38422647 10.1016/j.jpba.2024.116054

[16] A. J. Krotulski , D. C. Mata , C. R. Smith , et al., “Advances in Analytical Methodologies for Detecting Novel Psychoactive Substances: A Review,” Journal of Analytical Toxicology 49 (2025): 152–169. 39786399 10.1093/jat/bkae098

[17] B. P. Dos Santos , L. Birk , P. Schwarz , et al., “A Validated Dilute‐and‐Shoot LC–MS‐MS Urine Screening for the Analysis of 95 Illicit Drugs and Medicines: Insights From Clinical and Forensic Brazilian Cases,” Journal of Analytical Toxicology 48 (2024): 314–331. 38334744 10.1093/jat/bkae005

[18] K. Deventer , O. J. Pozo , A. G. Verstraete , and P. Van Eenoo , “Dilute‐and‐Shoot‐Liquid Chromatography‐Mass Spectrometry for Urine Analysis in Doping Control and Analytical Toxicology,” TrAC Trends in Analytical Chemistry 55 (2014): 1–13.

[19] G. Yang , S. Ge , R. Singh , et al., “Glucuronidation: Driving Factors and Their Impact on Glucuronide Disposition,” Drug Metabolism Reviews 49 (2017): 105–138. 28266877 10.1080/03602532.2017.1293682 PMC7660525

[20] X. Diao and M. A. Huestis , “New Synthetic Cannabinoids Metabolism and Strategies to Best Identify Optimal Marker Metabolites,” Frontiers in Chemistry 7 (2019): 109. 30886845 10.3389/fchem.2019.00109 PMC6409358

[21] M. Szeremeta , K. Pietrowska , A. Niemcunowicz‐Janica , A. Kretowski , and M. Ciborowski , “Applications of Metabolomics in Forensic Toxicology and Forensic Medicine,” International Journal of Molecular Sciences 22 (2021): 3010. 33809459 10.3390/ijms22063010 PMC8002074

[22] V. L. C. P. Bigão , B. R. B. Da Costa , J. J. M. Da Silva , B. S. De Martinis , and D. R. Tapia‐Blácido , “Use of Statistical Design of Experiments (DoE) in Forensic Analysis: A Tailored Review,” Forensic Chemistry 37 (2024): 100554.

[23] AAFS , “Standard Practices for Method Validation in Forensic Toxicology, ANSI/ASB Standard 036,” (2019).

[24] B. K. Matuszewski , M. L. Constanzer , and C. M. Chavez‐Eng , “Strategies for the Assessment of Matrix Effect in Quantitative Bioanalytical Methods Based on HPLC−MS/MS,” Analytical Chemistry 75 (2003): 3019–3030. 12964746 10.1021/ac020361s

[25] B. P. Dos Santos , L. Birk , P. De Souza Schwarz , S. Eller , T. F. De Oliveira , and M. D. Arbo , “A Comprehensive Analysis of Legislative Strategies for New Psychoactive Substances: The Brazilian Panorama,” Psychoactives 2 (2023): 242–255.

[26] T. B. Rodrigues , M. P. Souza , L. De Melo Barbosa , et al., “Synthetic Cannabinoid Receptor Agonists Profile in Infused Papers Seized in Brazilian Prisons,” Forensic Toxicology 40 (2022): 119–124. 36454481 10.1007/s11419-021-00586-7

[27] H. S. Bombana , G. De Paula Meirelles , R. A. De Oliveira , V. Leyton , and M. Yonamine , “Synthetic Cannabinoid Receptor Agonists in Oral Fluid: Development of a Dispersive Liquid–Liquid Microextraction Method With Liquid Chromatography–Mass Spectrometry Detection,” Journal of Analytical Toxicology (2025): bkaf027, 10.1093/jat/bkaf027. 40176730

[28] S. T. Ivory , J.‐A. Rotella , J. Schumann , and S. L. Greene , “A Cluster of 25B‐NBOH Poisonings Following Exposure to Powder Sold as Lysergic Acid Diethylamide (LSD),” Clinical Toxicology 60 (2022): 966–969. 35343858 10.1080/15563650.2022.2053150

[29] L. Birk , S. E. F. De Oliveira , G. Mafra , et al., “A Low‐Voltage Paper Spray Ionization QTOF‐MS Method for the Qualitative Analysis of NPS in Street Drug Blotter Samples,” Forensic Toxicology 38 (2020): 227–231.

[30] V. L. Meira , A. S. De Oliveira , L. S. A. Cohen , et al., “Chemical and Statistical Analyses of Blotter Paper Matrix Drugs Seized in the State of Rio de Janeiro,” Forensic Science International 318 (2021): 110588. 33278694 10.1016/j.forsciint.2020.110588

[31] B. De Souza Boff , J. Silveira Filho , K. Nonemacher , S. Driessen Schroeder , M. Dutra Arbo , and K. Z. Rezin , “New Psychoactive Substances (NPS) Prevalence Over LSD in Blotter Seized in State of Santa Catarina, Brazil: A Six‐Year Retrospective Study,” Forensic Science International 306 (2020): 110002. 31864775 10.1016/j.forsciint.2019.110002

[32] L. Glicksberg , K. Bryand , and S. Kerrigan , “Identification and Quantification of Synthetic Cathinones in Blood and Urine Using Liquid Chromatography‐Quadrupole/Time of Flight (LC‐Q/TOF) Mass Spectrometry,” Journal of Chromatography B 1035 (2016): 91–103. 10.1016/j.jchromb.2016.09.027 27697731

[33] L. Ambach , A. Hernández Redondo , S. König , V. Angerer , S. Schürch , and W. Weinmann , “Detection and Quantification of 56 New Psychoactive Substances in Whole Blood and Urine by LC–MS/MS,” Bioanalysis 7 (2015): 1119–1136. 26039809 10.4155/bio.15.48

[34] P. Kavanagh , A. Pechnikov , I. Nikolaev , G. Dowling , M. Kolosova , and A. Grigoryev , “Detection of ADB‐BUTINACA Metabolites in Human Urine, Blood, Kidney and Liver,” Journal of Analytical Toxicology 46 (2022): 641–650. 34341821 10.1093/jat/bkab088

[35] A. B. Godoi , N. D. J. Antunes , L. C. Rodrigues , A. F. Martins , and J. L. Costa , “In Vitro Metabolism and Metabolite Identification of Eutylone Using Rat Liver Microsomes,” Journal of Pharmaceutical and Biomedical Analysis 260 (2025): 116827. 40121735 10.1016/j.jpba.2025.116827

[36] K. N. Ellefsen , M. Concheiro , and M. A. Huestis , “Synthetic Cathinone Pharmacokinetics, Analytical Methods, and Toxicological Findings From Human Performance and Postmortem Cases,” Drug Metabolism Reviews 48 (2016): 237–265. 27249313 10.1080/03602532.2016.1188937

[37] A. T. Caspar , A. G. Helfer , J. A. Michely , et al., “Studies on the Metabolism and Toxicological Detection of the New Psychoactive Designer Drug 2‐(4‐Iodo‐2,5‐dimethoxyphenyl)‐N‐[(2‐methoxyphenyl)methyl]ethanamine (25I‐NBOMe) in Human and Rat Urine Using GC‐MS, LC‐MSn, and LC‐HR‐MS/MS,” Analytical and Bioanalytical Chemistry 407 (2015): 6697–6719. 26108532 10.1007/s00216-015-8828-6

[38] J. Xiang , D. Wen , W. Zhai , et al., “Metabolic Characterization of 25X‐NBOH and 25X‐NBOMe Phenethylamines Based on UHPLC‐Q‐Exactive Orbitrap MS in Human Liver Microsomes,” Journal of Pharmaceutical and Biomedical Analysis 242 (2024): 116020. 38359493 10.1016/j.jpba.2024.116020

[39] R. Pelletier , T. Gicquel , J. Carvelli , et al., “Severe 25E‐NBOH Intoxication Associated With MDPHP Intake: A Case Report, Metabolism Study, and Literature Review,” International Journal of Legal Medicine 138 (2024): 815–822. 38117418 10.1007/s00414-023-03151-6

[40] M. Nieddu , E. Baralla , F. Sodano , and G. Boatto , “Analysis of 2,5‐Dimethoxy‐Amphetamines and 2,5‐Dimethoxy‐Phenethylamines Aiming Their Determination in Biological Matrices: A Review,” Forensic Toxicology 41 (2023): 1–24. 36652064 10.1007/s11419-022-00638-6 PMC9849320

[41] S.‐Y. Fan , C.‐Z. Zang , P.‐H. Shih , et al., “A LC‐MS/MS Method for Determination of 73 Synthetic Cathinones and Related Metabolites in Urine,” Forensic Science International 315 (2020): 110429. 32784041 10.1016/j.forsciint.2020.110429

[42] C. Bouzoukas , P. Nikolaou , S. Athanaselis , A. Dona , C. Spiliopoulou , and I. Papoutsis , “Development, Validation and Applications of a GC/MS Method for the Simultaneous Determination of 9 Amphetamines and 12 NPS Analogues in Blood and Urine,” Forensic Science International 370 (2025): 112469. 40245655 10.1016/j.forsciint.2025.112469

[43] A. Pérez‐Alcaraz , F. Borrull , M. Calull , and C. Aguilar , “Cathinones in Urine Samples: A Review of Recent Advances for Their Determination by Chromatographic and Related Techniques,” TrAC Trends in Analytical Chemistry 143 (2021): 116347.

[44] L. A. Nisbet , F. M. Wylie , B. K. Logan , and K. S. Scott , “Gas Chromatography‐Mass Spectrometry Method for the Quantitative Identification of 23 New Psychoactive Substances in Blood and Urine,” Journal of Analytical Toxicology 43 (2019): 346–352. 30698723 10.1093/jat/bky109

[45] A. L. Fabris , R. Lanaro , J. L. Costa , and M. Yonamine , “Development of a Dispersive Liquid–Liquid Microextraction for Synthetic Cathinones in Biological Fluids Based on Principles of Green Analytical Toxicology,” Journal of Analytical Toxicology 47 (2023): 353–365. 36691915 10.1093/jat/bkad003

[46] P. Panuwet , R. E. Hunter, Jr. , P. E. D'Souza , et al., “Biological Matrix Effects in Quantitative Tandem Mass Spectrometry‐Based Analytical Methods: Advancing Biomonitoring,” Critical Reviews in Analytical Chemistry 46 (2016): 93–105. 25562585 10.1080/10408347.2014.980775 PMC4695332

[47] A. V. Eeckhaut , K. Lanckmans , S. Sarre , I. Smolders , and Y. Michotte , “Validation of Bioanalytical LC–MS/MS Assays: Evaluation of Matrix Effects,” Journal of Chromatography B 877 (2009): 2198–2207. 10.1016/j.jchromb.2009.01.003 19179125

[48] M. Jang , J. Kim , I. Han , and W. Yang , “Simultaneous Determination of LSD and 2‐Oxo‐3‐Hydroxy LSD in Hair and Urine by LC–MS/MS and Its Application to Forensic Cases,” Journal of Pharmaceutical and Biomedical Analysis 115 (2015): 138–143. 26188861 10.1016/j.jpba.2015.07.001

[49] P. C. Dolder , M. E. Liechti , and K. M. Rentsch , “Development and Validation of a Rapid Turboflow LC‐MS/MS Method for the Quantification of LSD and 2‐Oxo‐3‐Hydroxy LSD in Serum and Urine Samples of Emergency Toxicological Cases,” Analytical and Bioanalytical Chemistry 407 (2015): 1577–1584. 25542574 10.1007/s00216-014-8388-1

[50] R. Martin , J. Schürenkamp , A. Gasse , H. Pfeiffer , and H. Köhler , “Determination of Psilocin, Bufotenine, LSD and Its Metabolites in Serum, Plasma and Urine by SPE‐LC‐MS/MS,” International Journal of Legal Medicine 127 (2013): 593–601. 23183899 10.1007/s00414-012-0796-1

[51] D. Favretto , G. Frison , S. Maietti , and S. D. Ferrara , “LC‐ESI‐MS/MS on an Ion Trap for the Determination of LSD, iso‐LSD, nor‐LSD and 2‐Oxo‐3‐Hydroxy‐LSD in Blood, Urine and Vitreous Humor,” International Journal of Legal Medicine 121 (2007): 259–265. 16496170 10.1007/s00414-006-0078-x

[52] Y. Machado , J. Coelho Neto , R. A. Lordeiro , R. B. Alves , and E. Piccin , “Identification of New NBOH Drugs in Seized Blotter Papers: 25B‐NBOH, 25C‐NBOH, and 25E‐NBOH,” Forensic Toxicology 38 (2020): 203–215.

