# Supplementary material for: A Sensitive and Comprehensive LC‐MS/MS Method for the Analysis of Hallucinogens, Synthetic Cathinones, and Synthetic Cannabinoids in Urine Samples
Source: J Mass Spectrom. 2025 Sep 10;60(10):e5178. doi: 10.1002/jms.5178 (PMC12423360; doi:10.1002/jms.5178)
Supplement: Supplementary file 1 — Table S1: Complete matrix from fractional factorial design (25–1) for screening of significant variables. Table S2: Complete matrix from Doehlert design for solvent and sample volume optimization. Table S3: Complete matrix from simplex‐centroid design for extractor solvent optimization. Table S4: Processed sample stability results for each QC level. [file JMS-60-e5178-s001.docx]

**SUPPLEMENTARY MATERIAL**

**Table S1.** Complete matrix from fractional factorial design (2^5-1^) for screening of significant variables.

| **Run** | **Sample volume (μL)** | **Solvent volume (μL)** | **NaOH concentration (mol. L^-1^)** | **Salting out** | **Extraction time (s)** |
| --- | --- | --- | --- | --- | --- |
| 1 | 150 | 700 | 0.1 | -1 | 10 |
| 2 | 150 | 300 | 1.0 | -1 | 10 |
| 3 | 150 | 700 | 0.1 | +1 | 60 |
| 4 | 150 | 300 | 0.1 | +1 | 10 |
| 5 | 150 | 300 | 1.0 | +1 | 60 |
| 6 | 150 | 300 | 0.1 | -1 | 60 |
| 7 | 150 | 700 | 1.0 | -1 | 60 |
| 8 | 150 | 700 | 1.0 | +1 | 10 |
| 9 | 350 | 700 | 0.1 | -1 | 60 |
| 10 | 350 | 300 | 0.1 | -1 | 10 |
| 11 | 350 | 300 | 1.0 | +1 | 10 |
| 12 | 350 | 700 | 1.0 | -1 | 10 |
| 13 | 350 | 700 | 1.0 | +1 | 60 |
| 14 | 350 | 300 | 1.0 | -1 | 60 |
| 15 | 350 | 300 | 0.1 | +1 | 60 |
| 16 | 350 | 700 | 0.1 | +1 | 10 |

**Table S2.** Complete matrix from Doehlert design for solvent and sample volume optimization.

| **Run** | **Sample volume (μL)** | **Solvent volume (μL)** |
| --- | --- | --- |
| 1 | 100 | 500 |
| 2 | 175 | 200 |
| 3 | 175 | 800 |
| 4 | 250 | 500 |
| 5 | 250 | 500 |
| 6 | 250 | 500 |
| 7 | 325 | 200 |
| 8 | 325 | 800 |
| 9 | 400 | 500 |

**Table S3.** Complete matrix from simplex-centroid design for extractor solvent optimization.

| **Run** | **Ethyl acetate**  **(%, *v/v*)** | **Hexane**  **(%, *v/v*)** | **MTBE**  **(%, *v/v*)** |
| --- | --- | --- | --- |
| 1 | 100 | 0 | 0 |
| 2 | 0 | 100 | 0 |
| 3 | 0 | 0 | 100 |
| 4 | 50 | 50 | 0 |
| 5 | 50 | 0 | 50 |
| 6 | 0 | 50 | 50 |
| 7 | 66 | 17 | 17 |
| 8 | 17 | 66 | 17 |
| 9 | 17 | 17 | 66 |
| 10 | 33.3 | 33.3 | 33.3 |

**Table S4.** Processed sample stability results for each QC level.

| **Analyte** | **Processed sample stability (%)** | | |
| --- | --- | --- | --- |
|  | **Low QC**  **(*n* = 6)** | **Medium QC**  **(*n* = 6)** | **High QC**  **(*n* = 6)** |
| **Hallucinogens** |  |  |  |
| 2C-E | 99.1 | 99.2 | 101.2 |
| 25B-NBOH | 114.3 | 110.4 | 97.0 |
| 25B-NBOMe | 106.5 | 102.9 | 98.0 |
| 25C-NBOMe | 111.4 | 111.0 | 111.6 |
| 25E-NBOH | 122.1 | 121.2 | 109.7 |
| 25I-NBOH | 94.7 | 106.0 | 101.1 |
| 25I-NBOMe | 81.9 | 77.4 | 83.8 |
| 2-oxo-3-OH-LSD | 79.0 | 73.2 | 79.2 |
| LSD | 142.3 | 111.9 | 104.5 |
| **Synthetic cannabinoids** |  |  |  |
| ADB-BUTINACA | 100.9 | 110.7 | 99.6 |
| ADB-FUBINACA | 82.5 | 95.5 | 90.9 |
| **Synthetic cathinones** |  |  |  |
| 4-CDC | 105.9 | 107.8 | 95.2 |
| Dipentylone | 91.2 | 89.8 | 88.1 |
| Ethylone | 102.3 | 100.6 | 95.4 |
| Eutylone | 119.0 | 108.9 | 107.5 |
| Mephedrone | 109.3 | 108.6 | 100.9 |
| Methylone | 110.7 | 107.0 | 95.8 |
| N-butylpentylone | 126.4 | 122.8 | 119.6 |
| N-ethylheptedrone | 102.7 | 115.6 | 106.5 |
| Pentylone | 125.7 | 123.6 | 117.2 |
